# Relationship between pulse pressure and body mass index in active-duty Royal Thai Army personnel in Thailand

**DOI:** 10.1186/s12872-023-03390-w

**Published:** 2023-07-18

**Authors:** Boonsub Sakboonyarat, Jaturon Poovieng, Tanatip Sangkool, Sethapong Lertsakulbunlue, Kanlaya Jongcherdchootrakul, Phutsapong Srisawat, Mathirut Mungthin, Ram Rangsin

**Affiliations:** 1 Department of Military and Community Medicine, Phramongkutklao College of Medicine, Bangkok, 10400 Thailand; 2 Department of Medicine, Phramongkutklao College of Medicine, Bangkok, 10400 Thailand; 3 Department of Pharmacology, Phramongkutklao College of Medicine, Bangkok, 10400 Thailand; 4 Department of Parasitology, Phramongkutklao College of Medicine, Bangkok, 10400 Thailand

**Keywords:** Pulse pressure, Body mass index, High blood pressure, Royal Thai Army, Thailand

## Abstract

**Background:**

Elevated pulse pressure (PP) is a robust independent predictor of cardiovascular diseases. The relationship between PP and body mass index (BMI) was presented in a few studies. However, the findings were inconsistent. Therefore, the aim of the present study is to identify the association between elevated PP and BMI using a large sample of active-duty Royal Thai Army (RTA) personnel.

**Methods:**

A cross-sectional study was conducted through the use of the dataset obtained from the annual health examination database of RTA personnel in Thailand in 2022. BMI 25.0–29.9 kg/m^2^ was classified as obesity I, whereas BMI ≥ 30.0 kg/m^2^ was classified as obesity II. Elevated PP was defined as PP ≥ 50 mmHg. Multivariable linear regression and log-binomial regression models were utilized for determining the association between elevated PP and BMI.

**Results:**

A total of 62,113 active-duty RTA personnel were included in the study. The average BMI was 25.4 ± 3.8 kg/m^2^, while the average PP was 50.1 ± 11.2 mmHg. Compared to individuals with normal weight, the $\beta$ coefficients of PP and BMI were 1.38 (95% CI: 1.15–1.60) and 2.57 (95% CI: 2.25–2.88) in individuals with obesity I and obesity II, respectively. Effect modification by high blood pressure (BP) on the association between elevated PP and BMI was observed. Among participants with normal BP, in comparison with BMI of 18.5–22.9 kg/m^2^, the adjusted prevalence ratio (PR) for elevated PP was 1.23 (95% CI: 1.19–1.28) and 1.41 (95% CI: 1.35–1.48) in those with obesity I and obesity II, respectively. Meanwhile, among individuals with high BP, the adjusted PR for elevated PP was 1.05 (95% CI: 1.01–1.08) and 1.09 (95% CI: 1.06–1.13) in those with obesity I and obesity II, respectively.

**Conclusion:**

PP was positively associated with BMI in active-duty RTA personnel. High BP was the modifier of the association between PP and BMI. A weaker association between elevated PP and BMI was observed among RTA personnel with high BP.

**Supplementary Information:**

The online version contains supplementary material available at 10.1186/s12872-023-03390-w.

## Background

Cardiovascular diseases (CVD), including ischemic heart disease and stroke, are a leading cause of mortality in Thailand [1, 2]. The robust evidence demonstrated that high blood pressure (BP) is the independent risk factor for CVD [3–5]. High BP affects more than 30% of adults worldwide [6]. Meanwhile, in Thailand, adults aged 15 years and older suffer from high BP, approximately 25% in 2019 [7]. In addition to high BP, pulse pressure (PP) is the independent predictor of CVD, sudden cardiac death [8–11], and other comorbidities, including diabetes and its complications [12–14]. In Thailand, achieved BP control and lifestyle risk factor modification are the main recommendations other than pharmacological treatment to alleviate the CVD risk in the future, while PP was ignored [15].

At present, obesity, the metabolic risk factor for CVD, is also a health burden in the Thai population, in both civilian and military personnel [7, 16, 17]. Obesity prevalence among Thai civilians increased by approximately 30% over three decades [7, 18]. Meanwhile, the study on Royal Thai Army (RTA) personnel revealed that obesity prevalence increased from 42.1% in 2017 to 44.2% in 2021 [19]. Recently, a related study on RTA personnel emphasized that the prevalence of intermediate-to-high predicted 10-year risk for CVD surged from 24.9% in 2017 to 29.5 in 2021 [20]. High BP and high body mass index (BMI) are crucial in facilitating this trend [19, 20].

A recent nationwide study in Thailand provided information on the prevalence of elevated PP among patients with type 2 diabetes, which was approximately 50%. The study also indicated elevated PP was associated with diabetic retinopathy. [13]. However, there was no data on PP among RTA personnel. Moreover, a few studies demonstrated the relationship between PP and BMI in various populations except in Thailand, such as older adults [21], nondiabetic people [22], and the general population [23]. However, the findings in those studies were inconsistent. For instance, a study in the US among older adults with isolated systolic hypertension (HTN) who were not receiving antihypertensive medication declared a positive relationship between PP and BMI among patients with BMI over 30 kg/m^2^. In comparison, it was negatively associated among those with BMI $\le$ 30 kg/m^2^ [21]. Another study in Korea demonstrated that PP decreased with increasing BMI in patients with HTN [23]. However, a related study investigated by Wang et al. demonstrated no apparent association between BMI and high PP prevalence in those aged ≥ 60 years or with high BP [22].

In 2022, approximately 60,000 active-duty RTA personnel aged at least 35 years participated in health examinations provided by the RTA Medical Department (RTAMED). As mentioned, raised BMI and high BP were still major health issues among this population [19, 20, 24]. Thus, our objective was to adopt an extensive database of physical health examinations of RTA personnel in order to identify the association between PP and BMI. Furthermore, we explored the effect of measure modification of high BP on the relationship between elevated PP and BMI.

## Methods

### Study design and subjects

**Fig. 1:**
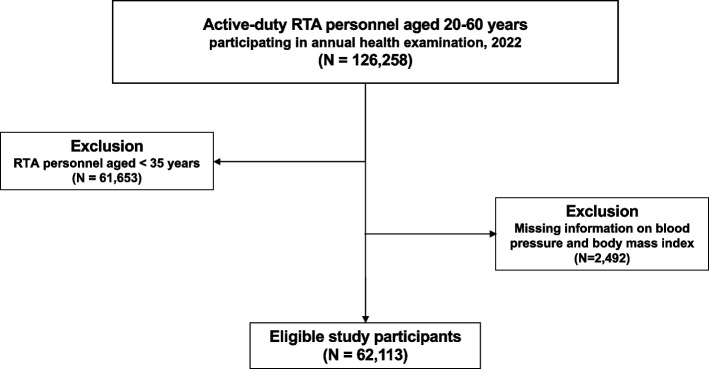
Flowchart of study

A cross-sectional study was carried out through the use of the dataset obtained from the annual health examination database of RTA personnel in 2022 from the RTAMED in Bangkok, Thailand. The RTAMED provides health examinations for active-duty RTA personnel through the Army Institute of Pathology (AIP), the Armed Forces Research Institute of Medical Sciences (AFRIMS), and 37 RTA hospitals nationwide (11 hospitals in the central, ten in the northeast, ten in the north, and six in the south) [19]. Typically, laboratory testing, comprising fasting plasma glucose (FPG) and total cholesterol (TC), would be provided for RTA personnel aged 35 years and older. Consequently, in order to identify the association between PP and BMI, the independent predictor of interest, we examined active-duty RTA personnel aged 35–60 years who participated in the annual health examination in 2022 (Fig. 1).

### Data collection

At the health examination session, a self-report using a standardized case report form was conducted to obtain characteristics data and behavioral factors, including age, sex, exercise, alcohol use, and smoking status. Lifestyle data were based on the data from the self-report questionnaire. Regular exercise was defined as exercise for 30 min/day and at least three days/week [25]. Current alcohol use was defined as having a history of consuming alcohol within 12 months [26]. A current smoker is defined as one who currently smokes cigarettes [27]. Furthermore, the dataset obtained from the annual health examination database of RTA personnel comprised systolic blood pressure (SBP) and diastolic blood pressure (DBP), body weight, and height. A trained operator managed anthropometric measurements. Laboratory test results, including FPG and TC, were also obtained from the health examination database.

The details of blood pressure (BP) measurement in the health examination of RTA personnel were published by Sakboonyarat et al. [24]. BP was measured through the use of an automatic blood pressure monitor by an operator trained in the standardized technique following the Thai guidelines on hypertension treatment [15]. The participants were advised to avoid caffeine and smoking for at least 30 min before measurement. Talking was not permitted during the measurement. Participants were instructed to be stationary for at least five minutes in a chair, with their feet on the floor and their arms supported at heart level [15]. Two measurements were taken, and the average was recorded. Regarding the various RTA hospitals nationwide providing the health examination session, we explored intraclass correlations (ICC) to demonstrate BP variance among hospitals. About 3% and 4% of the variability in SBP and DBP among RTA personnel can be explained by or attributed to the hospitals providing the health examination (Supplementary Table 1).

### The outcome, exposure, and covariates

Pulse pressure (PP) was calculated as follows: SBP – DBP [28]. Regarding the previous related study, elevated PP ≥ 50 mmHg were associated with all-cause, CVD, coronary heart disease, and cerebrovascular mortality [29]. Moreover, the normal distribution of PP in the present study with the average level at 50 mmHg; therefore, we categorized elevated PP using the cutoff point as PP ≥ 50 mmHg. Body mass index (BMI) was calculated by weight (in kg) divided by height (in meter-squared). BMI was classified according to the Asia–Pacific BMI classifications into five groups as follows: 18.5–22.9 kg/m^2^ (normal weight), < 18.5 kg/m^2^ (underweight), 23.0–24.9 kg/m^2^ (overweight), 25.0–29.0 kg/m^2^ (obesity I), and ≥ 30.0 kg/m^2^ (obesity II) [30]. High BP was defined as SBP ≥ 140 or DBP ≥ 90 mmHg [15]. High FPG was defined as FPG ≥ 126 mg/dL, whereas high TC was defined as TC ≥ 200 mg/dl [31]. The geographic regions of residence included Bangkok and the central, northeast, north, and south regions. The health insurance scheme covering study participants comprises civil servant medical benefits (CSMB), social security (SS), and universal health coverage (UHC). Smoking status was divided into three groups: never, ex-smoker, and current smoker. Alcohol use was also categorized into three groups: never, ex-drinker, and current alcohol use.

### Statistical analysis

Baseline characteristics were calculated through the use of descriptive statistics. Categorical variables were presented as percentages, while continuous variables were presented as mean, standard deviation (SD), median, and interquartile range (Q1–Q3). We explored the data distribution of PP, and the normal distribution was observed (Supplementary Table 2). We then categorized the elevated PP at the average point as PP ≥ 50 mmHg. The relationship between characteristics and PP was assessed. The association between the means of PP across baseline characteristics was compared using Student’s *t*-test or analysis of variance (ANOVA) as appropriate. At the same time, the *Chi*-square test was employed to compare the prevalence of high PP ($\ge$ 50 mmHg) across baseline characteristics.

In order to evaluate the association between PP and BMI, the assumptions associated with a linear regression model were tested. Linear regression analysis was then utilized for estimating the $\beta$ coefficient and 95% confidence intervals (CI). Furthermore, we used log-binomial regression to estimate the prevalence ratio (PR) and 95% CI in order to identify the association between elevated PP and BMI.

We conducted multivariable analysis in order to adjust the potential confounders. We included the variables associated with the outcome of interest in the final model, including sex, age, region, regular exercise, smoking status, alcohol use, high TC, high FPG, and high BP. We utilized margins command to illustrate the average adjusted prediction of PP mean and prevalence of elevated PP. Moreover, the interaction was tested to explore whether high BP modifies the relationship between high PP and BMI. We then identified the association between PP and BMI among individuals with normal BP and among those with high BP. 

A two-sided *p*-value less than 0.05 was considered statistically significant. Statistical analyses were conducted using StataCorp. 2021. *Stata Statistical Software: Release 17*. College Station, TX: StataCorp LLC, USA.

## Sensitivity analysis

In the current study, most participants were males (88.0%) to provide more information on the consistent association between BMI and PP among both sexes. Sex-specific differences in the association between BMI and PP were explored using linear regression and log-binomial regression analysis. Although the potential confounders were adjusted in the final model, the residual confounding effect may exist. Therefore, we then conducted a sensitivity analysis for unmeasured confounding using E-values estimated by the evalue package [32].

## Ethics considerations

The present study was reviewed and approved by the Institutional Review Board, Royal Thai Army Medical Department (Approval S056h/65), following the international guidelines, entailing the Declaration of Helsinki, the Belmont Report, the Council for International Organizations of Medical Sciences Guidelines, and the International Conference on Harmonization of Technical Requirements for Registration of Pharmaceuticals for Human Use-Good Clinical Practice (ICH-GCP). As a result of utilizing secondary data, a waiver of documentation of informed consent was obtained. An informed consent waiver was approved by the Institutional Review Board of the Royal Thai Army Medical Department.

## Results

### Characteristics of study participants

Table 1 presents the characteristics of 62,113 active-duty RTA personnel included in the study population. The majority of the study participants (88.0%) were males. Their mean age was 46.1 ± 7.8 years. One-third of RTA personnel came from the central region, while 96.9% were under the CSMB scheme. The mean BMI was 25.4 ± 3.8 kg/m^2^. Mean SBP and DBP were 130.6 ± 16.6 mmHg and 80.5 ± 11.5 mmHg, respectively. Mean PP was 50.1 ± 11.2 mmHg, while the median and IQR of PP were 49.0 (43.0–56.0) mmHg. Nearly two-thirds (58.9%) of study participants were current drinkers, while approximately one-fourth (28.3%) were current smokers.

**Table 1:** Characteristics of study participants

Characteristics	Total	Normal blood pressure^†^	High blood pressure^†^
	**n (%)**	**n (%)**	**n (%)**
**N**	62,113	42,995	19,118
**Sex**			
Men	54,662 (88.0)	36,837 (85.7)	17,825 (93.2)
Women	7,451 (12.0)	6,158 (14.3)	1,293 (6.8)
**Age (years)**			
35–39	17,526 (28.2)	13,473 (31.3)	4,053 (21.2)
40–44	11,369 (18.3)	8,307 (19.3)	3,062 (16.0)
45–49	11,064 (17.8)	7,488 (17.4)	3,576 (18.7)
50–54	9,351 (15.1)	6,014 (14.0)	3,337 (17.5)
55–60	12,803 (20.6)	7,713 (17.9)	5,090 (26.6)
mean ± SD	46.1 ± 7.8		
Median (Q1-Q3)	46.0 (39.0–53.0)		
**Regions**			
Bangkok	12,953 (20.9)	10,129 (23.6)	2,824 (14.8)
Central	20,084 (32.3)	13,480 (31.4)	6,604 (34.5)
Northeast	11,196 (18.0)	6,873 (16.0)	4,323 (22.6)
North	10,277 (16.5)	6,870 (16.0)	3,407 (17.8)
South	7,603 (12.2)	5,643 (13.1)	1,960 (10.3)
**Health insurance scheme**			
Civil servant medical benefit	58,619 (96.9)	40,389 (96.8)	18,230 (97.2)
Social security	1,388 (2.3)	1,056 (2.5)	332 (1.8)
Universal health coverage	481 (0.8)	291 (0.7)	190 (1.0)
**Body mass index, kg/m**^**2**^			
mean ± SD	25.4 ± 3.8	24.9 ± 3.6	26.6 ± 4.0
median (Q1-Q3)	25.0 (22.9–27.5)	24.5 (22.5–26.9)	26.0 (23.9–28.7)
**Systolic blood pressure, mmHg**			
mean ± SD	130.6 ± 16.6	122.7 ± 10.7	148.3 ± 13.4
median (Q1-Q3)	130.0 (120.0–139.0)	124.0 (116.0–131.0)	146.0 (140.0–155.0)
**Diastolic blood pressure, mmHg**			
mean ± SD	80.5 ± 11.5	75.5 ± 8.2	91.7 ± 9.9
median (Q1-Q3)	80.0 (73.0–88.0)	76.0 (70.0–82.0)	91.0 (86.0–97.0)
**Pulse pressure, mmHg**			
mean ± SD	50.1 ± 11.2	47.2 ± 8.7	56.6 ± 13.4
median (Q1-Q3)	49.0 (43.0–56.0)	47.0 (41.0–53.0)	56.0 (47.0–65.0)
**Fasting plasma glucose, mg/dL**			
mean ± SD	102.9 ± 35.1	100.4 ± 32.8	108.5 ± 39.3
median (Q1-Q3)	94.0 (87.0–104.0)	93.0 (87.0–102.0)	98.0 (90.0–110.0)
**Total cholesterol, mg/dL**			
mean ± SD	214.5 ± 49.3	214.0 ± 48.1	215.7 ± 52.1
median (Q1-Q3)	212.0 (184.0–242.0)	212.0 (184.0–241.0)	213.0 (184.0–243.0)
**Regular exercise**			
Yes	32,878 (53.8)	19,249 (45.5)	9029 (47.9)
No	28,278 (46.2)	23,048 (54.5)	9830 (52.1)
**Smoking status**			
Never	33,810 (54.7)	23,804 (55.6)	10,006 (52.6)
Ex-smoker	10,511 (17.0)	7117 (16.6)	3394 (17.9)
Current smoker	17,476 (28.3)	11,867 (27.7)	5609 (29.5)
**Alcohol use**			
Never	16,777 (27.1)	12,238 (28.6)	4539 (23.9)
Ex-drinker	8,610 (13.9)	6234 (14.6)	2376 (12.5)
Current drinker	36,443 (58.9)	24,333 (56.8)	12,110 (63.7)

High blood pressure (systolic blood pressure $\ge$ 140 mmHg or diastolic blood pressure $\ge$ 90 mmHg)

### Relationship between baseline characteristics and pulse pressure

Table 2 displays the relationship between baseline characteristics and PP. The difference in the mean of PP across BMI categories was observed (*p*-value < 0.001). Moreover, the prevalence of elevated PP ($\ge$ 50 mmHg) was more likely to be higher with high BMI *(p*-value < 0.001). Among RTA personnel with high BP, the prevalence of elevated PP was 69.7%. Nevertheless, it was 39.7% among those with normal BP (*p*-value < 0.001).

**Table 2:** Relationship between characteristics and pulse pressure

Variables	PP (mmHg)	*p*-value	PP $<$ 50 mmHg	PP $\ge$ 50 mmHg	*p*-value*
	**mean ± SD**		**n (%)**	**n (%)**	
**Body mass index, kg/m**^**2**^		< 0.001^§^			< 0.001
18.5–22.9	48.2 ± 11.0		9,062 (58.6)	6,400 (41.4)	
< 18.5	46.7 ± 11.8		486 (64.8)	264 (35.2)	
23.0–24.9	49.7 ± 10.9		8,122 (52.9)	7,224 (47.1)	
25.0.29.9	50.9 ± 11.0		11,212 (47.7)	12,307 (52.3)	
≥ 30.0	53.0 ± 11.9		2,858 (40.6)	4,178 (59.4)	
**Sex**		< 0.001^†^			< 0.001
Men	50.2 ± 11.1		27,737 (50.7)	26,925 (49.3)	
Women	49.3 ± 11.9		4,003 (53.7)	3,448 (46.3)	
**Age (years)**		< 0.001^§^			< 0.001
35–39	48.3 ± 10.2		10,066 (57.4)	7,460 (42.6)	
40–44	48.4 ± 10.3		6,485 (57.0)	4,884 (43.0)	
45–49	49.1 ± 10.7		6,041 (54.6)	5,023 (45.4)	
50–54	51.3 ± 11.6		4,374 (46.8)	4,977 (53.2)	
55–60	54.0 ± 12.4		4,774 (37.3)	8,029 (62.7)	
**Regions**		< 0.001^§^			< 0.001
Bangkok	49.9 ± 10.4		6,764 (52.2)	6,189 (47.8)	
Central	50.5 ± 11.0		9,911 (49.3)	10,173 (50.7)	
Northeast	52.8 ± 12.6		4,834 (43.2)	6,362 (56.8)	
North	47.1 ± 11.3		6,150 (59.8)	4,127 (40.2)	
South	49.5 ± 10.0		4,081 (53.7)	3,522 (46.3)	
**Health insurance scheme**		0.116^§^			0.184
Civil servant medical benefit	50.1 ± 11.2		29,839 (50.9)	28,780 (49.1)	
Social security	50.7 ± 12.2		675 (48.6)	713 (51.4)	
Universal health coverage	49.6 ± 10.8		253 (52.6)	228 (47.4)	
**Blood pressure, mmHg**		< 0.001^†^			< 0.001
SBP < 140 and DBP < 90	47.2 ± 8.7		25,947 (60.4)	17,048 (39.7)	
SBP ≥ 140 or DBP ≥ 90	56.6 ± 13.4		5,793 (30.3)	13,325 (69.7)	
**Fasting plasma glucose, mg/dL**		< 0.001^†^			< 0.001
< 126	49.8 ± 10.7		24,955 (51.9)	23,144 (48.1)	
≥ 126	53.1 ± 12.3		2,202 (41.6)	3,099 (58.5)	
**Total cholesterol, mg/dL**		< 0.001^†^			0.002
< 200	50.1 ± 11.3		11,188 (50.9)	10,797 (49.1)	
≥ 200	49.7 ± 10.9		18,546 (52.2)	16,960 (47.8)	
**Regular exercise**		< 0.001^†^			< 0.001
Yes	50.4 ± 11.3		14,806 (52.4)	13,472 (47.6)	
No	49.8 ± 11.2		16,401 (49.9)	16,477 (50.1)	
**Smoking status**		0.063^§^			0.002
Never	50.1 ± 11.4		17,179 (50.8)	16,631 (49.2)	
Ex-smoker	50.3 ± 11.0		5,293 (50.4)	5,218 (49.6)	
Current smoker	49.9 ± 11.0		9,128 (52.2)	8,348 (47.8)	
**Alcohol use**		< 0.001^§^			< 0.001
Never	50.0 ± 11.5		8,598 (51.2)	8,179 (48.8)	
Ex-drinker	48.6 ± 11.2		4,874 (56.6)	3,736 (43.4)	
Current drinker	50.5 ± 11.1		18,138 (49.8)	18,305 (50.2)	

^§^ANOVA, ^†^student *t*-test, *Chi-square, *PP* Pulse pressure (mmHg)

### Linear regression model to determine the association between PP and BMI

Table 3 shows a multivariable linear regression analysis of PP and BMI. A positive relationship between PP and BMI was observed among overall participants and those with normal and high BP (*p*-value < 0.001). Additionally, when BMI was further evaluated as categories, in comparison with the reference group (BMI 18.5–22.9 kg/m^2^), the adjusted $\beta$ coefficients for PP were 1.38 (95% CI: 1.15–1.60) and 2.57 (95% CI: 2.26–2.88) in overall participants with BMI 25.0–29.9 kg/m^2^ and BMI $\ge$ 30.0 kg/m^2^, respectively.

**Table 3:** Univariable and multivariable linear regression for the association between pulse pressure and body mass index

Variables	Pulse pressure (mmHg)					
	**Overall**^a^		**Normal blood pressure**^b^		**High blood pressure**^**c**^	
	${\varvec{\beta}}$ **coefficient (95% CI)**	***p-v*alue**	${\varvec{\beta}}$ **coefficient (95% CI)**	***p-v*alue**	${\varvec{\beta}}$ **coefficient (95% CI)**	***p-v*alue**
**Body mass index, kg/m**^**2**^						
Unadjusted model	0.42 (0.40, 0.45)	< 0.001	0.27 (0.25, 0.30)	< 0.001	0.09 (0.04, 0.14)	< 0.001
Adjusted model^**c**^	0.23 (0.21, 0.25)	< 0.001	0.28 (0.25, 0.30)	< 0.001	0.16 (0.12, 0.21)	< 0.001
**Body mass index category, kg/m**^**2**^						
Unadjusted model						
18.5–22.9	Ref		Ref		Ref	
< 18.5	-1.45 (-2.26, -0.64)	< 0.001	-1.44 (-2.13, -0.75)	< 0.001	0.84 (-1.59, 3.27)	0.497
23.0–24.9	1.52 (1.27, 1.76)	< 0.001	1.02 (0.80, 1.24)	< 0.001	0.02 (-0.60, 0.65)	0.939
25.0.29.9	2.71 (2.49, 2.94)	< 0.001	1.78 (1.58, 1.99)	< 0.001	-0.27 (-0.82, 0.29)	0.345
≥ 30.0	4.88 (4.56, 5.19)	< 0.001	2.81 (2.49, 3.13)	< 0.001	1.00 (0.34, 1.65)	0.003
Adjusted model^**c**^						
18.5–22.9	Ref		Ref		Ref	
< 18.5	-1.25 (-2.05, -0.46)	0.002	-1.55 (-2.28, -0.82)	< 0.001	1.08 (-1.47, 3.63)	0.407
23.0–24.9	0.83 (0.59, 1.07)	< 0.001	0.89 (0.66, 1.12)	< 0.001	0.41 (-0.23, 1.05)	0.211
25.0.29.9	1.38 (1.15, 1.60)	< 0.001	1.77 (1.55, 1.99)	< 0.001	0.43 (-0.14, 1.00)	0.141
≥ 30.0	2.57 (2.26, 2.88)	< 0.001	2.89 (2.55, 3.23)	< 0.001	1.96 (1.28, 2.65)	< 0.001

High blood pressure (systolic blood pressure $\ge$ 140 mmHg or diastolic blood pressure $\ge$ 90 mmHg)

^a^Adjusting for sex, age, regions, regular exercise, smoking status, alcohol use, high total cholesterol, high fasting plasma glucose, and high blood pressure

^b^Adjusting for sex, age, regions, regular exercise, smoking status, alcohol use, high total cholesterol, and high fasting plasma glucose

^c^*P* for interaction (high blood pressure and BMI) < 0.05

**Fig. 2:**
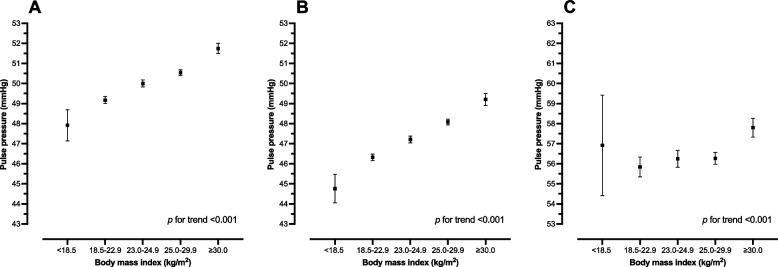
Average adjusted prediction of mean pulse pressure (mmHg) and 95% CI, by body mass index categories. **A** Average adjusted prediction of mean pulse pressure among overall RTA personnel, adjusting for high blood pressure, sex, age, regions, regular exercise, smoking status, alcohol use, high total cholesterol, high fasting plasma glucose. **B** Average adjusted prediction of mean pulse pressure among RTA personnel with normal blood pressure, adjusting for sex, age, regions, regular exercise, smoking status, alcohol use, high total cholesterol, high fasting plasma glucose. **C** Average adjusted prediction of mean pulse pressure among RTA personnel with high blood pressure, adjusting for sex, age, regions, regular exercise, smoking status, alcohol use, high total cholesterol, high fasting plasma glucose

Effect modification by high BP on the association between PP and BMI was noticed. Among participants with normal BP, the adjusted $\beta$ coefficients for PP were 1.77 (95% CI: 1.55–1.99) and 2.89 (95% CI: 2.55–3.23) in those with BMI 25.0–29.9 kg/m^2^ and BMI $\ge$ 30.0 kg/m^2^, respectively. A weaker association between PP and BMI categories was proved for participants with high BP. However, among individuals with high BP, in comparison with BMI 18.5–22.9 kg/m^2^, the adjusted $\beta$ coefficients for PP were 0.43 (95% CI: -0.14–1.00) and 1.96 (95% CI: 1.28–2.65) in those with BMI 25.0–29.9 kg/m^2^ and BMI $\ge$ 30.0 kg/m^2^, respectively. Figure 2 illustrates the average adjusted prediction of mean PP by BMI categories. Dose–response relationship of higher PP with higher BMI categories was observed among overall participants and individuals with normal BP.

### Log-binomial regression model to determine the association between elevated PP and BMI

The association between elevated PP and BMI was analyzed through the use of multivariable log-binomial regression (Table 4). After adjusting for the potential confounders, the association between elevated PP and BMI was observed. Among overall participants, the prevalence of elevated PP was more likely to be higher (adjusted PR: 1.15; 95% CI: 1.13–1.18) among RTA personnel with BMI 25.0–29.9 kg/m^2^ compared to those with BMI 18.5–22.9 kg/m^2^. Similarly, the adjusted PR for elevated PP of those with BMI $\ge$ 30.0 kg/m^2^ was 1.26 (95% CI: 1.22–1.30).

**Table 4:** Univariable and multivariable log-binomial regression for the association between elevated pulse pressure ($\ge$ 50 mmHg) and body mass index

Variables	Elevated pulse pressure ($\ge$ 50 mmHg)					
	**Overall**^a^		**Normal blood pressure**^b^		**High blood pressure**^b^	
	**PR (95% CI)**	***p-v*alue**	**PR (95% CI)**	***p-v*alue**	**PR (95% CI)**	***p-v*alue**
**Body mass index, kg/m**^**2**^						
Unadjusted model	1.03 (1.03–1.03)	< 0.001	1.03 (1.03–1.03)	< 0.001	1.01 (1.01–1.01)	< 0.001
Adjusted model^c^	1.03 (1.03–1.04)	< 0.001	1.05 (1.04–1.06)	< 0.001	1.01 (1.01–1.02)	< 0.001
**Body mass index category, kg/m**^**2**^						
Unadjusted model						
18.5–22.9	Ref		Ref		Ref	
< 18.5	0.85 (0.77–0.94)	0.001	0.84 (0.74–0.95)	0.006	0.97 (0.86–1.11)	0.684
23.0–24.9	1.14 (1.11–1.17)	< 0.001	1.12 (1.09–1.16)	< 0.001	1.00 (0.97–1.03)	0.948
25.0.29.9	1.26 (1.24–1.29)	< 0.001	1.23 (1.20–1.27)	< 0.001	1.01 (0.99–1.04)	0.335
≥ 30.0	1.43 (1.40–1.47)	< 0.001	1.38 (1.33–1.44)	< 0.001	1.04 (1.01–1.08)	0.007
Adjusted model^c^						
18.5–22.9	Ref		Ref		Ref	
< 18.5	0.88 (0.80–0.97)	0.010	0.82 (0.72–0.94)	0.005	1.03 (0.90–1.18)	0.686
23.0–24.9	1.08 (1.05–1.11)	< 0.001	1.10 (1.07–1.15)	< 0.001	1.02 (0.99–1.06)	0.171
25.0.29.9	1.15 (1.13–1.18)	< 0.001	1.23 (1.19–1.28)	< 0.001	1.05 (1.01–1.08)	0.004
≥ 30.0	1.26 (1.22–1.30)	< 0.001	1.41 (1.35–1.48)	< 0.001	1.09 (1.06–1.13)	< 0.001

*PR* Prevalence ratio, High blood pressure (systolic blood pressure $\ge$ 140 mmHg or diastolic blood pressure $\ge$ 90 mmHg)

^a^Adjusting for sex, age, regions, regular exercise, smoking status, alcohol use, high total cholesterol, high fasting plasma glucose, and high blood pressure

^b^Adjusting for sex, age, regions, regular exercise, smoking status, alcohol use, high total cholesterol, and high fasting plasma glucose

^c^*P* for interaction (high blood pressure and BMI) < 0.05

**Fig. 3:**
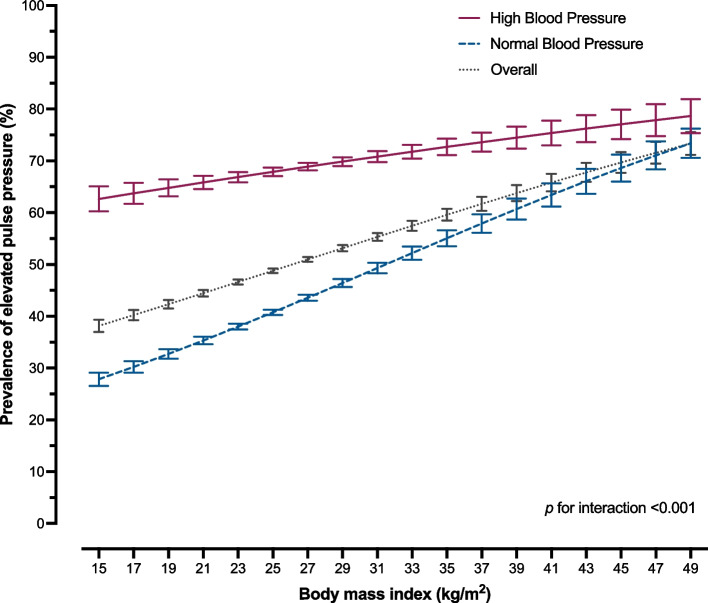
Spline curve of body mass index for the average adjusted prediction of prevalence of elevated pulse pressure ($\ge$ 50 mmHg) and 95% CI, by blood pressure groups

**Fig. 4:**
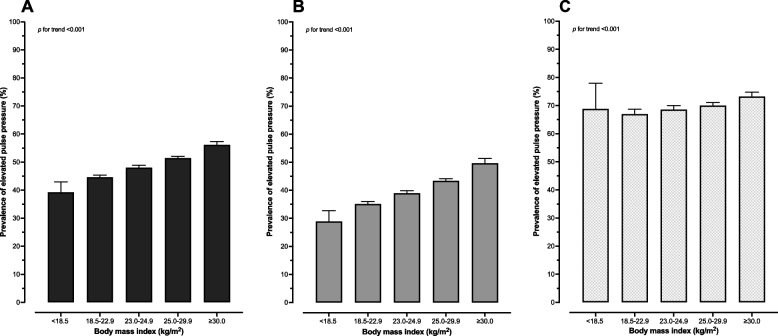
Average adjusted prediction of the prevalence of elevated pulse pressure ($\ge$ 50 mmHg) and 95% CI, by body mass index categories. **A** Average adjusted prediction of prevalence of elevated pulse pressure ($\ge$ 50 mmHg) among overall RTA personnel, adjusting for sex, age, regions, regular exercise, smoking status, alcohol use, high total cholesterol, high fasting plasma glucose, and high blood pressure. **B** Average adjusted prediction of prevalence of elevated pulse pressure ($\ge$ 50 mmHg) among RTA personnel with normal blood pressure, adjusting for sex, age, regions, regular exercise, smoking status, alcohol use, high total cholesterol, and high fasting plasma glucose. **C** Average adjusted prediction of prevalence of elevated pulse pressure ($\ge$ 50 mmHg) among RTA personnel with high blood pressure, adjusting for sex, age, regions, regular exercise, smoking status, alcohol use, high total cholesterol, and high fasting plasma glucose

Furthermore, effect modification by high BP on the association between elevated PP and BMI was discovered (Fig. 3). Among participants with normal BP, the adjusted PR for elevated PP was 1.23 (95% CI: 1.19–1.28) and 1.41 (95% CI: 1.35–1.48) in those with BMI 25.0–29.9 kg/m^2^ and BMI $\ge$ 30.0 kg/m^2^, respectively. A weaker association between elevated PP and BMI categories was recognized among participants with high BP. In comparison with BMI 18.5–22.9 kg/m^2^, the adjusted PR for elevated PP was 1.05 (95% CI: 1.01–1.08) and 1.09 (95% CI: 1.06–1.13) in those with BMI 25.0–29.9 kg/m^2^ and BMI $\ge$ 30.0 kg/m^2^, respectively. Figure 4 illustrates the average adjusted prediction of the elevated PP prevalence by BMI categories. Dose–response relationship of rising prevalence of elevated PP with higher BMI categories was demonstrated among overall participants and individuals with normal BP.

Regarding the sensitivity analysis, after adjusting for potential confounders, the dose–response relationship between PP and BMI category was observed in males and females (Supplementary Tables 3 and 4). The E-value for the PR to identify the association between the unmeasured confounder and the primary predictor (BMI) and outcome (PP) is presented in Supplementary Table 5.

## Discussion

The relationship between PP and BMI was identified in active-duty RTA personnel in Thailand through the use of an extensive database of the physical health examinations of the RTA personnel in 2022. After controlling potential confounders, a positive association between PP and BMI was detected. Moreover, the effect of measure modification of high BP on the association between PP and BMI was observed. Furthermore, it was found that the prevalence of elevated PP was higher in RTA personnel with obesity I and obesity II compared to those with normal weight in overall study participants, individuals with normal BP, and those with high BP. To the best of our knowledge, this is the first and largest study examining the relationship between elevated PP and higher BMI in the Thai population.

Recently, a large serial cross-sectional study among RTA personnel in Thailand revealed that the prevalence of intermediate-to-high predicted 10-year risk for CVD remarkably surged from 24.9% in 2017 to 29.5 in 2021 [20]. Elevated SBP and high BMI among RTA personnel are crucial in promoting this trend [19, 20]. At present, achieved BP control and lifestyle risk factor modification are the main recommendations other than pharmacological treatment to attenuate the CVD risk in the future, while PP was ignored [15]. However, in line with the existing literature, elevated PP is a strong independent predictor of CVD, including myocardial infarction, stroke, heart failure, and sudden cardiac death [8, 10, 33, 34]. Hence, our findings highlighted that elevated PP should be emphasized among individuals with normal or high BP.

### Comparison with previous studies

We found an independent positive association of PP with increasing BMI among RTA personnel aged 35–60 years. In addition, the sex-specific association between PP and BMI in both men and women was also observed. Moreover, we observed a dose–response relationship with a relatively precise association between elevated PP and raised BMI. After adjusting for covariates, we discovered that the prevalence of elevated PP among participants with overweight, obesity I, and obesity II was estimated to be 8%, 15%, and 26% higher than those with normal weight. This finding is compatible with a recent study in China, reporting that BMI was linearly and positively related to PP [22]. On the contrary, a related study on US adults aged 55 years and older revealed that PP continued to decrease as BMI increased until the BMI exceeded 30.1 kg/m^2^. However, in the study on US older adults, alcohol use and physical exercise which may be potential confounders were not included in the final model [21]. Unfortunately, our study implicates only active-duty RTA personnel. We did not have the opportunity to assess the relationship between PP and BMI among the older age groups.

In the present study, a substantially higher prevalence of elevated PP was observed among participants with high BP compared to those with normal BP. Furthermore, the effect modification of high BP on the association between elevated PP and BMI was observed. Among individuals with normal BP, the prevalence of elevated PP among individuals with overweight, obesity I, and obesity II was estimated to be 10%, 23%, and 41% higher than those with normal weight. At the same time, a weaker association between elevated PP and BMI was observed among participants with high BP. Our finding demonstrated that the prevalence of elevated PP among individuals with overweight, obesity I, and obesity II was estimated to be 2%, 5%, and 9% higher than those with normal weight. Regarding the robust evidence, PP and arterial stiffness are strongly correlated [11], while high BP is related to lower arterial elasticity, resulting in arterial stiffness [35, 36]. Thus, among individuals with high BP, low arterial elasticity is likely to exist, and arterial stiffness is prone to develop. This phenomenon may dilute the association between elevated PP and BMI among participants with high BP, which is weaker than that in individuals with normal BP.

Similarly, a related study in China found a weak association between elevated PP and BMI among participants with high BP [22]. Nevertheless, another study in Korea [23] reported that PP increased with high BMI among people with normal BP but decreased with high BMI among individuals with hypertension. However, we noticed that, in that study [23], smoking status, alcohol usage, and physical exercise were not adjusted in the final model.

### Potential mechanisms

The association between elevated PP and higher BMI or obesity may be explained by several mechanisms. Firstly, obesity results in increased sympathetic nervous system activity, which plays a key role in contributing to the reduction in vasodilatation and compliance, facilitating arterial stiffness [37–39]. Secondly, adipocyte-derived factor, the metabolic alteration in adipose tissue in the setting of obesity, results in an elevated circulating level of adipocyte-derived inflammatory cytokines, enclosing tumor necrosis factor-α, interleukin-6, angiotensinogen, and aldosterone-stimulating factors [38]. Furthermore, it can boost the recruitment and activation of proinflammatory immune cells in the vasculature, contributing to the development of arterial stiffness [38–40]. Another explanation is that metabolic demand rises among individuals with higher BMI. Therefore, increased cardiac output is one of the mechanisms to meet this high metabolic demand. However, the resting heart rate of individuals with obesity does not grow significantly. Thus, stroke volume has to increase more to raise cardiac output, consequently reducing arterial compliance [22, 37].

### Limitations

The present study has several limitations. First, this was a cross-sectional study. The causal relationship between exposure and outcome could not be presented. Second, regarding the secondary database used, the information on any medication use, including antihypertensive medications and weight loss medication, possibly affecting BP and BMI, was not collected.

Third, typically, physical health examination sessions for RTA personnel provided laboratory testing for RTA personnel aged 35 years and older. Consequently, we encircled only active-duty personnel aged 35–60 years, and we did not have the opportunity to assess the association between elevated PP and BMI among individuals aged less than 35 and more than 60 years. Therefore, the generalizability of the results may be limited. Additionally, the sex distribution of study participants comprised a greater proportion of male participants, which may limit the generalizability to other populations. However, the results reflected an actual situation in this study population. Furthermore, the consistent results from sensitivity analysis indicated a positive association between PP and BMI among male and female participants.

Forth, the information on BP was obtained from the data of health examinations performed by various RTA hospitals nationwide, which may have variability among hospitals. However, ICC demonstrated that only 3–4% of the variability in BP among study participants could be explained by or attributed to the hospitals providing the health examination. Fifth, the behavioral data, including smoking status, alcohol use, and exercise information, were based on self-reported data, which may be subject to recall or social desirability bias, affecting the results.

Finally, we did not have the opportunity to collect information on dietary patterns. Hence, some unmeasured confounders were not included in the adjusted model. However, the evidence for causality from these E-values looks relatively weak because insubstantial unmeasured confounding would be needed to decrease the observed association.

### Further directions

The repeated measure of BP may contribute to the robustness of the association of PP and BMI. Additionally, further longitudinal studies should be conducted to provide definitive evidence on the causal relationship between PP and BMI. Our results from linear and log-binomial regression models provided robust evidence of the association between raised PP and higher BMI in individuals with normal and high BP. The results highlighted that obesity was an independent predictor for elevated PP. Recently, weight reduction is recommended to attenuate BMI and also reduce BP [16, 19, 20, 37]. Furthermore, the previous study indicated that diet and exercise intervention could reduce BMI, reducing PP [37]. According to our findings and existing literature [37], weight reduction should be considered in RTA personnel with both normal BP and high BP, which may also lead to a reduction in PP, a potential predictor of CVD. Thus, regarding feasibility and straightforwardness, in addition to SBP and DBP, PP should be evaluated and paid attention to in clinical practice.

Additionally, we recommended weight management and BP control among RTA with high BP through a modifiable lifestyle other than pharmacological treatment. For instance, during the annual health examination session, RTA personnel with higher BMI, especially over 25.0 kg/m^2^, should be provided counseling to modify lifestyle risks, such as regular exercise and a healthy diet [41, 42]. At the same time, RTA personnel with high BP should be encouraged to have close follow-ups with medical doctors, improve medication adherence[24] and be advised to reduce or stop smoking for current smokers [43].

## Conclusion

In conclusion, increased PP was positively associated with higher BMI in active-duty RTA personnel. The prevalence of elevated PP was higher in RTA personnel with obesity compared to those with normal weight in overall study participants, those with normal BP, and those with high BP. High BP was the modifier of the association between PP and BMI. A weaker association between elevated PP and BMI was depicted among participants with high BP.

## Supplementary Information

**Additional file 1:** **Supplementary Table 1.** Intraclass correlation (ICC) demonstrates blood pressure, and body mass index  variance due to hospitals (providing the health examination; n=39) by total variance. **Supplementary Table 2.** Data distribution and dispersion of pulse pressure.  **Supplementary Table 3.** Univariable and multivariable linear regression for the association between pulse pressure and body mass index, stratified by sex. 

## Data Availability

The data that support the findings of this study are available from the Royal Thai Army Medical Department, but restrictions apply to the availability of these data, which were used under license for the current study, and so are not publicly available. Data are however available from the authors upon reasonable request and with permission of the Royal Thai Army Medical Department (contact Boonsub Sakboonyarat via boonsub1991@pcm.ac.th).

## Abbreviations

BP: Blood pressure
SBP: Systolic blood pressure
DBP: Diastolic blood pressure
PP: Pulse pressure
BMI: Body mass index
TC: Total cholesterol
FPG: Fasting plasma glucose
APR: Adjusted prevalence ratio
CI: Confidence interval
SD: Standard deviation
