# Supplementary material for: Relationship between pulse pressure and body mass index in active-duty Royal Thai Army personnel in Thailand
Source: BMC Cardiovasc Disord. 2023 Jul 18;23:361. doi: 10.1186/s12872-023-03390-w (PMC10355046; doi:10.1186/s12872-023-03390-w)
Supplement: Supplementary file 1 — Additional file 1: Supplementary Table 1. Intraclass correlation (ICC) demonstrates blood pressure, and body mass index variance due to hospitals (providing the health examination; n=39) by total variance. Supplementary Table 2. Data distribution and dispersion of pulse pressure. Supplementary Table 3. Univariable and multivariable linear regression for the association between pulse pressure and body mass index, stratified by sex. [file 12872_2023_3390_MOESM1_ESM.pdf]

Appendix

Relationship between pulse pressure and body mass index in active-duty Royal Thai Army personnel in Thailand

Boonsub Sakboonyarat[1], Jaturon Poovieng[2], Tanatip Sangkool[1], Sethapong Lertsakulbunlue[3], Kanlaya Jongcherdchootrakul[1], Phutsapong Srisawat[1], Mathirut Mungthin[4], *Ram Rangsin[1]

[1]Department of Military and Community Medicine, Phramongkutklao College of Medicine, Bangkok 10400, Thailand
[2]Department of Medicine, Phramongkutklao College of Medicine, Bangkok 10400, Thailand
[3]Department of Pharmacology, Phramongkutklao College of Medicine, Bangkok 10400, Thailand
[4]Department of Parasitology, Phramongkutklao College of Medicine, Bangkok 10400, Thailand

Table of Contents

Table S1. Intraclass correlation (ICC) demonstrates blood pressure variance due to hospitals (providing the health examination) by total variance.	Page 3
Table S2. Data distribution and dispersion of pulse pressure	Page 4
Table S3. Sex-specific univariable and multivariable linear regression for the association between pulse pressure and body mass index	Page 5
Table S4. Sex-specific univariable and multivariable log-binomial regression for the association between elevated pulse pressure (≥50 mmHg) and body mass index	Page 6
Table S5. Sensitivity analysis for unmeasured confounding using E-value for prevalence ratio.	Page 7

Supplementary Table 1. Intraclass correlation (ICC) demonstrates blood pressure, and body mass index variance due to hospitals (providing the health examination; n=39) by total variance.

Blood pressure	Systolic blood pressure	Diastolic blood pressure	Body mass index
**Total N**	62,113	62,113	62,113
**Groups**	39	39	39
**Avg. no. per group**	1,592.6	1,592.6	1,592.6
**Variance (constant)**	9.22	5.63	.05
**Variance (residual)**	262.60	129.33	14.51
**ICC (SE)**	0.03 (0.01)	0.04 (0.01)	0.004 (0.001)
**95 % CI of ICC**	0.02-0.05	0.02-0.05	0.002-0.006

SE: standard error, CI: confidence interval

Approximately 3% of the variability in systolic blood pressure among RTA personnel can be explained by or attributed to the hospitals providing the health examination.

Approximately 4% of the variability in diastolic blood pressure among RTA personnel can be explained by or attributed to the hospitals providing the health examination.

Approximately 0.4% of the variability in body mass index among RTA personnel can be explained by or attributed to the hospitals providing the health examination.

Supplementary Table 2. Data distribution and dispersion of pulse pressure

Measurement	Pulse pressure
**Total N**	62,113
**Mean**	50.09
**Standard deviation**	11.21
**Median**	49.00
**Min-Max**	20.00-110.00
**Q1-Q3**	43.00-56.00
**Skewness**	0.68
**Kurtosis**	4.35

Supplementary Table 3. Univariable and multivariable linear regression for the association between pulse pressure and body mass index, stratified by sex.

Variables	Pulse pressure (mmHg)					
	Overall§		Men†		Women†	
	***β* coefficient (95% CI)**	***p-value***	***β* coefficient (95% CI)**	***p-value***	***β* coefficient (95% CI)**	***p-value***
**Body mass index, kg/m^2**						
Unadjusted model	0.42 (0.40, 0.45)	<0.001	0.39 (0.37, 0.42)	<0.001	0.59 (0.53, 0.65)	<0.001
Adjusted model*	0.23 (0.21, 0.25)	<0.001	0.40 (0.37, 0.42)	<0.001	0.47 (0.41, 0.54)	<0.001
**Body mass index category, kg/m^2**						
Unadjusted model						
18.5-22.9	Ref.		Ref.		Ref.	
<18.5	-1.45 (-2.26, -0.64)	<0.001	-1.33 (-2.34, -0.33)	0.009	-0.86 (-2.31, 0.59)	0.245
23.0-24.9	1.52 (1.27, 1.76)	<0.001	1.22 (0.95, 1.49)	<0.001	2.72 (1.99, 3.44)	<0.001
25.0.29.9	2.71 (2.49, 2.94)	<0.001	2.33 (2.09, 2.57)	<0.001	4.83 (4.16, 5.49)	<0.001
≥30.0	4.88 (4.56, 5.19)	<0.001	4.46 (4.13, 4.80)	<0.001	6.99 (6.09, 7.90)	<0.001
Adjusted model*						
18.5-22.9	Ref.		Ref.		Ref.	
<18.5	-1.25 (-2.05, -0.46)	0.002	-1.41 (-2.39, -0.42)	0.005	-0.02 (-1.39, 1.35)	0.976
23.0-24.9	0.83 (0.59, 1.07)	<0.001	0.69 (0.43, 0.95)	<0.001	0.91 (0.21, 1.61)	0.010
25.0.29.9	1.38 (1.15, 1.60)	<0.001	1.15 (0.92, 1.39)	<0.001	2.23 (1.57, 2.88)	<0.001
≥30.0	2.57 (2.26, 2.88)	<0.001	2.37 (2.03, 2.70)	<0.001	2.85 (1.94, 3.75)	<0.001

§Adjusting for sex, age, regions, regular exercise, smoking status, alcohol use, high total cholesterol, high fasting plasma glucose, and high blood pressure
†Adjusting for age, regions, regular exercise, smoking status, alcohol use, high total cholesterol, high fasting plasma glucose, and high blood pressure
**P* for interaction (Sex and Body mass index) < 0.05

Supplementary Table 4. Univariable and multivariable log-binomial regression for the association between elevated pulse pressure (≥50 mmHg) and body mass index, stratified by sex.

Variables	Elevated pulse pressure (≥50 mmHg)					
	Overall[§]		Men[†]		Women[†]	
	PR (95% CI)	*p-value*	PR (95% CI)	*p-value*	PR (95% CI)	*p-value*
**Body mass index, kg/m²**						
Unadjusted model	1.03 (1.03-1.03)	<0.001	1.05 (1.05-1.06)	<0.001	1.08 (1.07-1.09)	<0.001
Adjusted model*	1.03 (1.03-1.04)	<0.001	1.03 (1.03-1.04)	<0.001	1.03 (1.03-1.04)	<0.001
**Body mass index category, kg/m²**						
Unadjusted model						
18.5-22.9	Ref.		Ref.		Ref.	
<18.5	0.85 (0.77-0.94)	0.001	0.87 (0.77-0.98)	0.015	0.87 (0.72-1.04)	0.114
23.0-24.9	1.14 (1.11-1.17)	<0.001	1.11 (1.08-1.14)	<0.001	1.27 (1.18-1.36)	<0.001
25.0.29.9	1.26 (1.24-1.29)	<0.001	1.23 (1.20-1.26)	<0.001	1.48 (1.39-1.57)	<0.001
≥30.0	1.43 (1.40-1.47)	<0.001	1.40 (1.36-1.44)	<0.001	1.64 (1.53-1.76)	<0.001
Adjusted model*						
18.5-22.9	Ref.		Ref.		Ref.	
<18.5	0.88 (0.80-0.97)	0.010	0.87 (0.77-0.98)	0.020	0.98 (0.84-1.14)	0.801
23.0-24.9	1.08 (1.15-1.11)	<0.001	1.07 (1.04-1.09)	<0.001	1.10 (1.03-1.19)	0.008
25.0.29.9	1.15 (1.13-1.18)	<0.001	1.14 (1.11-1.16)	<0.001	1.21 (1.14-1.29)	<0.001
≥30.0	1.26 (1.22-1.30)	<0.001	1.24 (1.20-1.28)	<0.001	1.29 (1.18-1.40)	<0.001

[§]Adjusting for sex, age, regions, regular exercise, smoking status, alcohol use, high total cholesterol, high fasting plasma glucose, and high blood pressure
[†]Adjusting for age, regions, regular exercise, smoking status, alcohol use, high total cholesterol, high fasting plasma glucose, and high blood pressure
[*]*P* for interaction (Sex and Body mass index) < 0.05
PR: prevalence ratio

Supplementary Table 5. Sensitivity analysis for unmeasured confounding using E-value for prevalence ratio.

Variables	E-value for prevalence ratio	
	Point estimate	Confidence interval
**Overall§**		
Body mass index, kg/m^2	1.21	1.21
**Body mass index category, kg/m^2**		
18.5-22.9	Ref.	Ref.
<18.5	1.53	1.21
23.0-24.9	1.37	1.28
25.0.29.9	1.57	1.51
≥30.0	1.83	1.74
**Normal blood pressure†**		
Body mass index, kg/m^2	1.28	1.24
**Body mass index category, kg/m^2**		
18.5-22.9	Ref.	Ref.
<18.5	1.74	1.32
23.0-24.9	1.43	1.34
25.0.29.9	1.76	1.67
≥30.0	2.17	2.04
**High blood pressure†**		
Body mass index, kg/m^2	1.11	1.11
**Body mass index category, kg/m^2**		
18.5-22.9	Ref.	Ref.
<18.5	1.21	1.00
23.0-24.9	1.16	1.00
25.0.29.9	1.28	1.11
≥30.0	1.40	1.31
**Men***		
Body mass index, kg/m^2	1.21	1.21
**Body mass index category, kg/m^2**		
18.5-22.9	Ref.	Ref.
<18.5	1.57	1.17
23.0-24.9	1.34	1.22
25.0.29.9	1.54	1.46
≥30.0	1.79	1.69
**Women***		
Body mass index, kg/m^2	1.21	1.21
**Body mass index category, kg/m^2**		
18.5-22.9	Ref.	Ref.
<18.5	1.17	1.00
23.0-24.9	1.43	1.21
25.0.29.9	1.71	1.54
≥30.0	1.90	1.64

§Adjusting for sex, age, regions, regular exercise, smoking status, alcohol use, high total cholesterol, high fasting plasma glucose, and high blood pressure

†Adjusting for sex, age, regions, regular exercise, smoking status, alcohol use, high total cholesterol, and high fasting plasma glucose

* Adjusting for age, regions, regular exercise, smoking status, alcohol use, high total cholesterol, high fasting plasma glucose, and high blood pressure
